# Practice facilitation to implement alcohol-related care in Veterans Health Administration liver clinics: a study protocol

**DOI:** 10.1186/s43058-020-00062-0

**Published:** 2020-07-31

**Authors:** Madeline C. Frost, George N. Ioannou, Judith I. Tsui, E. Jennifer Edelman, Bryan J. Weiner, Olivia V. Fletcher, Emily C. Williams

**Affiliations:** 1 Health Services Research & Development (HSR&D) Center of Innovation for Veteran-Centered and Value-Driven Care, Veterans Affairs (VA) Puget Sound Health Care System, 1660 South Columbian Way, Seattle, WA 98108 USA; 2 Department of Health Services, University of Washington School of Public Health, 1959 NE Pacific St, Seattle, WA 98195 USA; 3 Department of Medicine, University of Washington School of Medicine, 325 9th Ave, Seattle, WA 98104 USA; 4 Yale Schools of Medicine and Public Health, 367 Cedar Street, ES Harkness, suite 401, New Haven, CT 06510 USA; 5 Department of Global Health, University of Washington School of Public Health, 1959 NE Pacific St, Seattle, WA 98195 USA

**Keywords:** Alcohol, Screening, Brief intervention, Alcohol use disorder treatment, Liver, Hepatology, Practice facilitation, Implementation

## Abstract

**Background:**

Alcohol-related care, including screening, brief intervention, and provision of/referral to medication or behavioral treatments for alcohol use disorder, could be delivered in liver clinics to better reach patients with chronic liver conditions. However, the provision of alcohol-related care in liver clinics is currently suboptimal. Practice facilitation is an evidence-based implementation strategy that may address barriers, harness facilitators, and optimize the implementation of alcohol-related care in liver clinic settings using a clinic-centered approach. We report the protocol of a study to test a practice facilitation intervention to implement alcohol-related care in four Veterans Health Administration liver clinics.

**Methods:**

This study will employ a Hybrid Type 3 effectiveness-implementation design, in which implementation outcomes are considered primary and clinical outcomes secondary. Intervention and evaluation design were informed by the Consolidated Framework for Implementation Research. Qualitative data collected from clinical stakeholders and patients were used to tailor the intervention. The intervention involves a 6-month period of external practice facilitation, including regular meetings to identify clinic goals, challenges, and solutions; engagement of clinic champions; provision of training and development of educational materials for clinic staff and patients; and performance monitoring and feedback. Ongoing formative evaluation involves the collection of quantitative facilitator tracking data and qualitative data from meeting notes and patient interviews to describe intervention acceptability, feasibility, and adoption, and adjust implementation as needed. In the summative evaluation, implementation outcomes (clinic rates of screening, brief intervention, and treatment referral/receipt) and clinical outcomes (unhealthy alcohol use, liver health) will be assessed among patients in participating clinics using secondary electronic health record data and interrupted time series analysis.

**Discussion:**

This will be the first study to our knowledge to test practice facilitation to implement alcohol-related care in liver clinic settings. Results from formative and summative evaluation will inform a framework for the successful implementation of effective alcohol-related care through practice facilitation in liver clinics, which may ultimately lead to better health outcomes for patients with chronic liver disease.

Contributions to the literature

- Alcohol-related care could be delivered in liver clinics to better reach patients with chronic liver conditions, but barriers to delivery of alcohol-related care in liver clinic settings are likely.
- Practice facilitation is an evidence-based implementation strategy that may address barriers, harness facilitators, and optimize implementation of alcohol-related care in liver clinic settings.
- This paper reports the protocol of the first study to our knowledge to test a practice facilitation intervention to implement alcohol-related care in liver clinics.
- The protocol will provide context for the eventual publication of study findings and useful information for researchers studying the implementation of alcohol-related care.

## Background

Alcohol use is a leading cause of global morbidity and mortality [1], and in the USA, alcohol-related deaths have increased substantially over the past two decades [2]. Deaths related to alcohol use were a major contributor to a decline in overall US life expectancy observed between 2015 and 2017 [3], and in 2017, almost one third of such deaths resulted from alcohol-related liver disease [4]. Unhealthy alcohol use is typically defined as a spectrum ranging from drinking above recommended limits to alcohol use disorder (AUD) [5]; however, for people with chronic liver conditions, even low levels of alcohol consumption may be harmful [6, 7].

There are a range of effective clinical care options available to address alcohol use. The US Preventive Services Task Force recommends screening for unhealthy alcohol use for all patients and the provision of brief intervention (advice to moderate use or abstain) for all patients who screen positive [8, 9]. Systematic reviews [10, 11] and evidence-based clinical guidelines [12–14] support multiple treatment options for AUD, including medications (including 3 Food and Drug Administration-approved and several off-label use medications) and behavioral interventions (e.g., cognitive-behavioral theory). However, only a minority of patients with AUD receive evidence-based treatment [15–18].

Liver clinics are important settings in which to provide effective alcohol-related care. Helping patients with alcohol-related and other liver conditions, including alcohol-associated hepatitis, hepatitis C virus (HCV) infection, cirrhosis, and hepatocellular carcinoma, to abstain from or minimize alcohol use is a critical component of improving their liver health, and experts have called for the provision of alcohol screening, brief intervention, and medication and behavioral AUD treatment for patients with chronic liver disease [6, 7]. As of 2019, the American Association for the Study of Liver Disease practice guidelines on the diagnosis and treatment of alcohol-associated liver diseases recommend routine alcohol screening, brief intervention, and referral to AUD treatment for patients in liver clinics [19]. Further, liver clinics may provide a prime opportunity to provide alcohol-related care as patients are focused on their liver health during the visit and often return for regular visits during which repeated interventions can be offered. In particular, the introduction of curative treatment for HCV has resulted in repeated visits to liver clinics for many patients with HCV, and, as alcohol use acts synergistically with HCV to increase risk of liver harm and mortality [6, 20–25], these visits are an optimal time to provide alcohol-related care.

Though liver clinics are well-suited to the provision of evidence-based alcohol-related care, the provision of such care is likely impacted by addressable barriers including lack of knowledge, lack of skills training, stigma, and logistical barriers [26–29]. Practice facilitation is an evidence-based strategy that may address barriers and optimize the implementation of alcohol-related care in liver clinic settings using a clinic-centered approach. Practice facilitation is a multi-level intervention in which a practice facilitator offers tools, resources, and hands-on guidance and content expertise to assist the team in developing strategies to address gaps in care and tailor workflow flexibly to the clinic setting. The goal is for the facilitator to support the clinic in strategizing how to best harness facilitators and address barriers at multiple levels, and to enable local teams to successfully implement evidence-based practices [30–33]. A key element of practice facilitation is the development of internal capacity within the clinic to create change, such that the evidence-based practice will continue after the facilitation intervention concludes [32, 33]. Studies have demonstrated the effectiveness of practice facilitation in primary care settings; a meta-analysis found that odds of providing evidence-based preventive care were 2.76 times higher (95% CI 2.18–3.43) in primary care settings with practice facilitation [30]. Practice facilitation is increasingly being tested as a means of increasing the provision of substance use-related care in diverse clinical settings [34], including alcohol-related care in primary care clinics [35]. However, to our knowledge, the use of practice facilitation to implement alcohol-related care in liver clinic settings has not yet been evaluated. This protocol describes the design and evaluation of a practice facilitation intervention intended to increase and improve the provision of evidence-based alcohol-related care in four Veterans Health Administration (VA) liver clinics.

## Methods

### Overall design

This study will examine the implementation and effectiveness of practice facilitation to improve the provision of evidence-based alcohol-related care in four VA liver clinics, using a Hybrid Type 3 effectiveness-implementation design [36] in which implementation outcomes are considered primary and clinical outcomes are considered secondary. Preliminary qualitative data were collected from 47 clinical stakeholders (including physicians, nurse practitioners, nurses, social workers, and other clinic staff) and 43 patients to inform the design and tailoring of the intervention. The intervention involves a 6-month period of external practice facilitation at each site, including regular meetings to identify clinic goals, challenges, and solutions; engagement of clinic champions; provision of training and development of educational materials for clinic staff and patients; and performance monitoring and feedback. Ongoing formative evaluation involves the collection of quantitative and qualitative data to describe the implementation of the intervention (acceptability, feasibility, adoption) and adjust implementation as needed. Implementation (penetration) and clinical (unhealthy alcohol use, liver health) outcomes will be assessed among patients in participating clinics using secondary electronic health record data and interrupted time series analysis.

### Conceptual framework

**Fig. 1:**
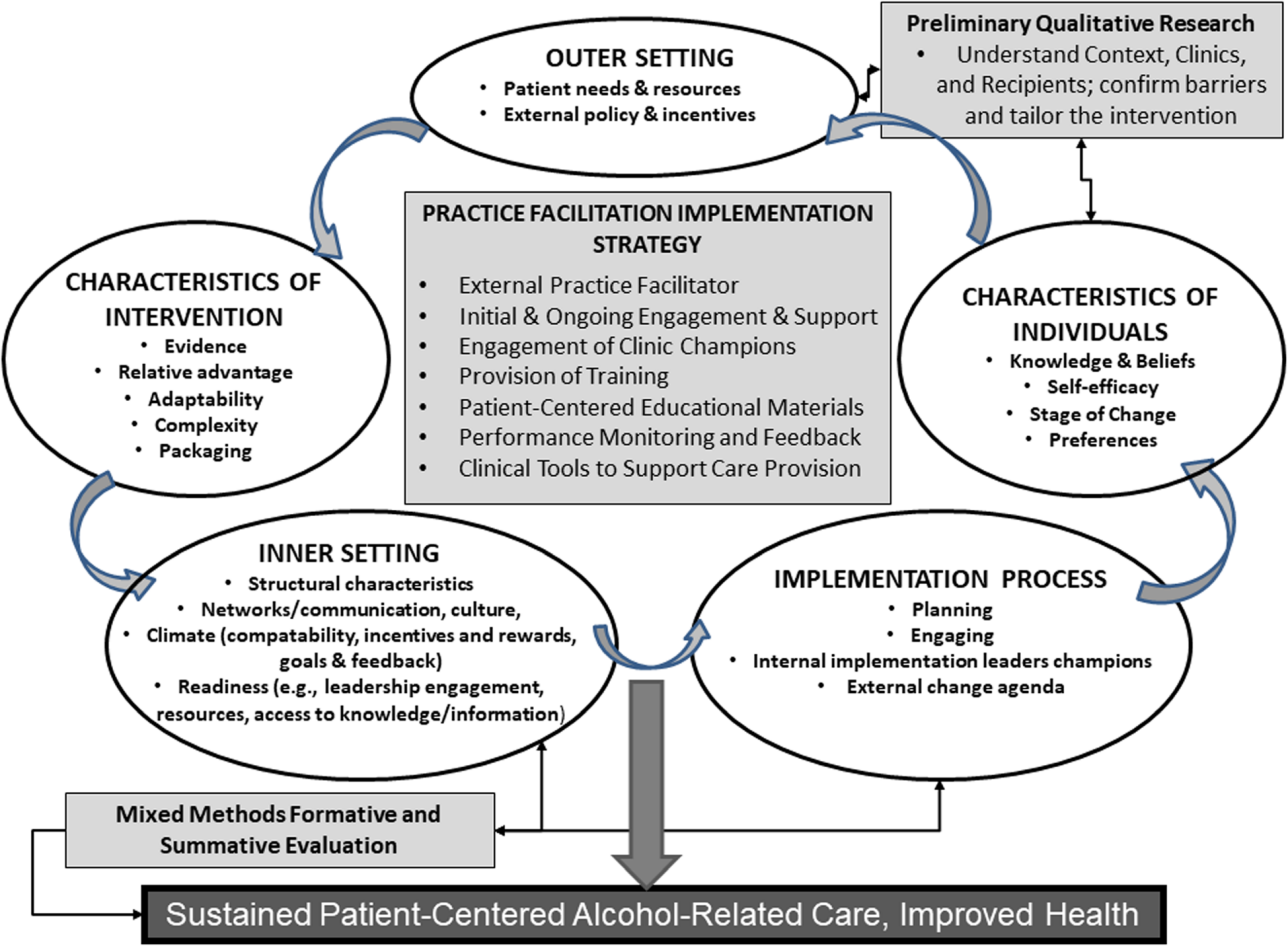
Research plan and implementation strategy guided by the Consolidated Framework for Implementation Research (CFIR)

The intervention and evaluation design were informed by the Consolidated Framework for Implementation Research (CFIR; Fig. 1). The CFIR includes five domains of implementation, each with multiple subdomains: (1) characteristics of the intervention, (2) outer setting, (3) inner setting, (4) characteristics of individuals, and (5) implementation process [37]. CFIR domains informed the development of interview questions for preliminary qualitative data collection with clinical stakeholders and patients, and guide the tailoring and ongoing implementation of practice facilitation. Additionally, the CFIR domains informed the definition of implementation and clinical outcomes included in the summative evaluation.

### Setting and participating sites

This study is conducted in four VA liver clinics located in the western United States. Alcohol-associated liver disease has contributed to increased midlife mortality among VA patients, mirroring trends in the general US population [3, 38]. VA has implemented electronic clinical reminders for annual alcohol screening for primary care patients using the Alcohol Use Disorders Identification Test Consumption (AUDIT-C) and brief intervention for patients who screen positive for unhealthy alcohol use [39, 40], but no such reminders exist for liver clinic providers. Sites were selected based on adequate numbers of patients with liver conditions and unhealthy alcohol use identified in preliminary research [41, 42], diversity of staffing models and patient populations, and existing clinical relationships across sites.

### Pre-intervention data collection and planning: tailoring the intervention

Preliminary qualitative data were collected from clinical stakeholders and patients to plan the intervention and tailor the practice facilitation approach for each participating clinic. Clinical stakeholders included all clinic staff who interact with patients and/or help the clinic to function, including clinic directors, physicians, nurse practitioners, nurses, physician assistants, pharmacists, social workers, fellows or trainees, and front desk staff. A total of 47 clinical stakeholders were interviewed, ranging from 8 to 14 at each clinic. The goal of clinical stakeholder interviews was to understand how the clinic functions, to gauge the clinic’s readiness to move forward different aspects of alcohol-related care, and to identify existing resources and areas in need of support. Patients with a past-year visit to a participating clinic with a documented AUDIT-C screen and a diagnosed liver condition were recruited for telephone-based qualitative interviews. Purposive sampling was used to achieve variation in the patient interview sample with respect to gender, race/ethnicity, level of alcohol use ranging from non-drinking to severe unhealthy alcohol use identified using AUDIT-C score, and liver condition diagnoses. A total of 41 patients were interviewed, ranging from 8 to 12 at each clinic. The goal of patient interviews was to understand patients’ experience receiving care in liver clinics, experience with and perspectives on alcohol-related care, and suggestions for how to best provide alcohol-related care in the liver clinic in order to promote the provision of patient-centered care through this practice facilitation intervention.

A rapid assessment process [43, 44] was used to analyze qualitative data and identify themes to help tailor the intervention. Specifically, interviews were digitally recorded and transcribed, and each interview transcript was then distilled into a 1- to 2-page template summarizing essential responses to each question and highlighting demonstrative quotations. Each template was reviewed for accuracy by a second team member. Templates were then distilled into key themes and presented to the entire investigative team for feedback and refinement. Feedback from the project team, summaries of key themes, and individual templates were used to plan facilitation strategies.

### Practice facilitation intervention

Intervention components are described in Fig. 1. Our model employs external facilitation [33, 45], which involves a single practice facilitator who is external to the clinic and provides support and expertise in facilitation, implementation, and alcohol-related care. The facilitator is part of a larger project team including the principal investigator and multiple interdisciplinary co-investigators with clinical and research experience and expertise in implementation science, qualitative and quantitative evaluation methods, liver health, and alcohol-related care. The team also includes two qualitative interviewers/analysts and a data manager.

At the beginning of the 6-month implementation period, the practice facilitator holds a “kickoff” meeting with the clinic to orient them to the project and to begin the facilitation process. At this meeting, the facilitator presents an aggregate summary of key findings from qualitative data that were collected during the pre-implementation phase to ensure the project team has correctly interpreted the findings, to fill in gaps in information, and to promote discussion among clinic staff about what they think the facilitation process should look like. Ongoing meetings are scheduled with clinic staff and the practice facilitator consistent with the needs and wants of the clinic. These meetings may be conducted in person or remotely depending on the location of each clinic. When possible, ongoing meetings are integrated into clinics’ existing team meetings or quality improvement meetings. The content of ongoing meetings depends on the progress of implementation—meeting time may be devoted to identifying problems and discussing possible solutions, deciding on courses of action, establishing smaller groups or identifying individuals to take on certain tasks, and/or presenting training/educational material.

One or more “clinic champions”—members of the clinic team who take on leadership roles and ownership of the implementation—are identified at each clinic [33]. Individuals who express enthusiasm and/or have relevant expertise and interest are engaged by the practice facilitator in additional one-on-one or small group meetings to move the implementation forward, and they develop goals and ideas that are brought back to the larger group for discussion in larger ongoing meetings. Clinic champions provide valuable insight, engage key stakeholders, and help get others in the clinic on board with plans. They may be focused on singular or multiple aspects of the intervention, depending on their knowledge, skills, and interests. How goals are addressed and in what order depends on the needs and resources of each clinic. In the beginning of the intervention, identifying goals that are prioritized by the clinic and are relatively easy to accomplish can help build trust and enthusiasm for the intervention. Flexibility and responsiveness to the clinic’s goals, barriers, and strengths is a key element of successful practice facilitation [33]. The practice facilitator aims to identify and move forward goals in collaboration with clinic champions in order to maximize stakeholder buy-in and effectiveness of strategies.

As goals are identified and addressed, the practice facilitator provides needed education related to unhealthy alcohol use and the provision of evidence-based alcohol-related care and may bring in external experts such as specialty addiction treatment providers or primary care providers who provide alcohol-related care to conduct trainings with liver clinic staff. The practice facilitator also develops written materials to support providers, such as flowcharts to guide them through the provision of alcohol-related care and/or pamphlets with information on alcohol use, liver health, and alcohol-related care for use with patients.

Finally, the practice facilitator develops strategies with the clinic for ongoing performance monitoring and feedback, in which key outcomes are measured and reported back to the clinic in monthly aggregate reports. Performance monitoring and feedback are supported by a data manager/analyst on the project team.

### Formative evaluation methods

Formative evaluation is a continuous process that involves collecting and reviewing qualitative and quantitative data from multiple sources throughout the implementation period [46]. In addition to providing a detailed and accurate description of the implementation, the formative evaluation process allows the project team to continually reflect on implementation progress, barriers, and facilitators, and to adjust the approach as needed throughout the implementation. Formative evaluation focuses on three implementation-related concepts: (1) acceptability, or the extent to which providing alcohol-related care in the clinic is agreeable, palatable or satisfactory to stakeholders; (2) feasibility, or the extent to which alcohol-related care can be successfully carried out within the clinic; and (3) adoption, or the initial level of update of alcohol-related care in the clinic.

The primary source of formative evaluation data is a weekly recurring meeting with the core project team (including the practice facilitator, principal investigator, and project managers/qualitative leads). In these meetings, the practice facilitator reports on all practice facilitation activities and progress made during the past week. The practice facilitator takes detailed notes throughout the week in preparation for reporting out to the group at these meetings. A project manager takes detailed meeting minutes, and the minutes are later coded in qualitative analysis software. Data are coded as barriers to or facilitators of implementation, and additional codes emerge from the data. Codes are organized under the CFIR domains.

Additionally, the practice facilitator carefully tracks all activities and tasks that are completed as part of the intervention and the amount of time spent on each task using an adapted version of the Facilitation Tracking Tool developed by the VA Quality Enhancement Research Initiative for Team-Based Behavioral Health [33]. This tracking system is designed to support formative evaluation and later to describe the relative importance of different intervention components and estimate the amount of time required from the practice facilitator to implement in each clinic.

Finally, after practice facilitation efforts end in each clinic, a limited number of interviews (5–10 per clinic) will be conducted with patients who had documented alcohol-related care in order to assess intervention fidelity with respect to patients’ perspectives. These interviews will assess patient-reported receipt of alcohol-related care in the clinic (screening, brief intervention, and receipt of AUD treatment medications and/or referral to specialty treatment as appropriate), shared decision-making (with the 9-item Shared Decision Making Questionnaire) [47], patient experience of care quality (with selected items from the Consumer Assessment of Healthcare Providers and Systems Clinician and Group Survey) [48], and open-ended questions about patients’ interactions with providers and suggestions to improve alcohol-related care.

### Summative evaluation methods

Summative evaluation outcomes will be measured using secondary data extracted from the VA Corporate Data Warehouse, a national repository of clinical and administrative data. The primary implementation outcomes will describe the penetration of alcohol-related care, operationalized as clinic rates of screening for unhealthy alcohol use among all clinic patients, receipt of brief intervention among patients with unhealthy alcohol use, receipt of AUD treatment medications, and/or referral to and receipt of specialty addictions treatment among patients with severe unhealthy alcohol use (e.g., AUDIT-C ≥ 8) or alcohol use disorder (defined as International Classification of Disease, 10th Revision, Clinical Modification codes for alcohol abuse or dependence, excluding in remission). Secondary clinical outcomes will include rates of clinic patients with unhealthy alcohol use, prevalence with documented alcohol use disorder, liver fibrosis tests (Fibrosis-4), and several HCV-specific outcomes (including rate of HCV treatment completion and rate of sustained virologic response among patients with HCV).

Summative evaluation outcomes will be assessed using an interrupted time series design, in which each clinic serves as its own control (pre/post-intervention). The study sample will include all patients who received VA care in one of the participating clinics during each month in the 24 months preceding, and 12 months following, implementation per clinic. Outcomes will be calculated as rates for each 1-month period in the 36-month study period. The denominator will be all patients attending the liver clinic during the time period. We will use an ordinary least squares regression model to determine whether there is a change in the level and trend over time (slope) of each outcome after dissemination of the implementation intervention, compared to before. The primary hypothesis for this study is that rates of alcohol-related care will increase in magnitude after the onset of the intervention. Further, we hypothesize that the slope will increase after the intervention compared with before the intervention. We will test our null hypotheses using an alpha of 0.05. We will conduct secondary analyses to assess the effect of the intervention in subsamples of patients, including those defined by demographic characteristics (e.g., racial/ethnic groups) and those identified with comorbid substance use (e.g., opioid use disorder).

### Statistical power

Power analysis was based on the estimation of the treatment effect at the interruption of the series. Assuming alpha = 0.05, two-tailed test, power = 0.90, 24 pre-intervention months, 12 post-intervention months, and 200 patients/month (estimated conservatively based on each clinic’s enrollment of at least 120 patients/month, with ~ 50% expected to have unhealthy alcohol use), we would have a minimum detectable effect size (Cohen’s *d*) of 0.21. Given an estimated baseline rate of 75% of patients receiving alcohol-related care and OR = exp(1.65 × *d*), this translates into 90% power to detect an average 8% increase in alcohol-related care (80% power for a 4% increase) [49].

### Study status

Preliminary qualitative interviews have been conducted with patients and clinical stakeholders, which included an in-person site visit at each participating clinic and telephone interviews with all patient participants. Preliminary qualitative data have been analyzed, and the study team has reviewed these findings to tailor the intervention to each clinic. The 6-month practice facilitation implementation phase is underway in the first clinic; however, implementation has been paused due to the COVID-19 pandemic, which has substantially impacted normal clinic operations and clinic staff availability. No data have been cleaned or analyzed for the formative or summative evaluation.

## Discussion

This protocol describes the design and evaluation of a practice facilitation intervention intended to increase and improve the provision of evidence-based alcohol-related care in four VA liver clinics. This will be the first study to our knowledge to test a practice facilitation intervention to implement alcohol-related care in liver clinic settings. If effective, this intervention may serve as a model for the implementation of alcohol-related care in liver clinics in the VA and in other health care systems. Increasing the provision of evidence-based alcohol-related care in liver clinic settings has the potential to improve patient outcomes and prevent alcohol-related morbidity and mortality in high-risk patients.

### Strengths and limitations

There are both strengths and limitations of this study. One strength is the use of stakeholder input to tailor the implementation intervention. Additionally, the study employs mixed methods to evaluate a range of formative evaluation outcomes and multiple summative evaluation outcomes. Generalizability may be limited by the inclusion of clinics with whom investigators had established relationships. Generalizability may also be somewhat limited to the VA health care system, though the flexibility of practice facilitation should allow it to be successfully adapted for implementation in other settings. Additionally, the quasi-experimental approach used to evaluate the intervention does not enable an unequivocal assessment of causality [49].

## Conclusion

Alcohol use is a leading cause of morbidity and mortality in the USA and globally [1, 2], and for people with chronic liver conditions, even low levels of alcohol consumption may be harmful [6, 7]. Liver clinics are important settings in which to provide evidence-based alcohol-related care; however, the provision of such care is likely impacted by multiple barriers. Practice facilitation is an evidence-based strategy that may address barriers and optimize the implementation of alcohol-related care in liver clinic settings using a clinic-centered approach. This will be the first study to our knowledge to test a practice facilitation intervention to implement alcohol-related care in liver clinic settings. Results from formative and summative evaluation will potentially provide support and a framework for the successful implementation of effective alcohol-related care through practice facilitation in liver clinics.

## Data Availability

Not applicable.

## Abbreviations

AUD: Alcohol use disorder
AUDIT-C: Alcohol Use Disorders Identification Test Consumption
CFIR: Consolidated Framework for Implementation Research
HCV: Hepatitis C virus
VA: Veterans Health Administration
